# EVERY DAY I HAVE THE BLUES

**DOI:** 10.1093/geroni/igad104.1750

**Published:** 2023-12-21

**Authors:** Elwood Blues

**Affiliations:** GSA Bo Diddley Track, Joliet, Illinois, United States

## Abstract

The Blues Brothers are an American blues and soul revivalist band founded in 1978 by comedians Dan Aykroyd and John Belushi as part of a musical sketch on Saturday Night Live. Belushi and Aykroyd fronted the band, in character, respectively, as lead vocalist ’Joliet’ Jake Blues and harmonica player/vocalist Elwood Blues, donning black suits with matching fedoras and sunglasses.

